# The work challenges faced by primary healthcare workers in Guangzhou during the COVID-19 pandemic prevention and control period of 2021–2022: a qualitative study

**DOI:** 10.3389/fpubh.2024.1454759

**Published:** 2024-10-24

**Authors:** Yuqing Jin, Huiyao Feng, Qin Xiao, Fenglin Tian, Huan Yao, Runquan Zhang, Qiqi Wu, Haiyuan Zhu, Wanshan Zheng, Jin Chen, Tao Liu, Wenjun Ma, Xiongfei Chen, Xiaomei Dong

**Affiliations:** ^1^Department of Public Health and Preventive Medicine, School of Medicine, Jinan University, Guangzhou, Guangdong, China; ^2^Development and Planning Department, Dongguan People's Hospital, Dongguan, Guangdong, China; ^3^Department of Primary Public Health, Guangzhou Center for Disease Control and Prevention, Guangzhou, Guangdong, China

**Keywords:** COVID-19, primary healthcare workers, work challenges, qualitative survey, public health emergency management

## Abstract

**Background:**

Primary healthcare workers in China were vital in controlling COVID-19, facing unprecedented challenges due to the pandemic’s suddenness and transmissibility. This paper analyzed their work in Guangzhou, highlighting key difficulties in past epidemic control efforts.

**Methods:**

Semi-structured qualitative interviews were conducted with selected primary healthcare workers in Guangzhou City and audio-recorded until saturation of themes was reached. The data were analyzed independently for themes using NVivo V.12.

**Results:**

Thematic analysis of qualitative interview data revealed that challenges and difficulties encountered in grassroots epidemic prevention and control work during the COVID-19 pandemic were primarily categorized into seven dimensions: human resource allocation, material and vehicle support, epidemic prevention subsidies and compensation, humanistic care, upper-level management, horizontal collaboration, and resident cooperation.

**Conclusion:**

Based on the findings, the research team recommends that administrative departments and institutions related to primary healthcare should appropriately schedule personnel work and rest time, adjust work content flexibly, optimize the allocation of medical and logistical resources, and increase investment in primary healthcare work as well as staff protection.

## 1 Introduction

Since the outbreak of the COVID-19 pandemic at the end of 2019, this global public health crisis had a far-reaching impact on the healthcare systems of various countries (1). Guangzhou, located in the southern part of China, is the capital city of Guangdong Province, a national central city, and a mega-city. It serves as a major transportation hub and a comprehensive transportation hub city, acting as the southern gateway for China’s connection with the world. With a high degree of urbanization, Guangzhou has a vast population of permanent and transient residents. Guangdong Province bore the greatest pressure of overseas COVID-19 importation cases during the pandemic, with nearly 30,000 people under quarantine daily. As the most important city in Guangdong Province, Guangzhou Baiyun International Airport handled a huge passenger flow. The large number of people under entry quarantine observation increased the risk of disease transmission during the pandemic. This necessitated a more proactive approach by the primary healthcare system in tracking, testing, and isolating cases (2). Primary healthcare institutions not only needed to maintain daily medical services, but also had to respond and adapt quickly to the new requirements of epidemic prevention and control (3).

In the normalization of COVID-19 prevention and control, primary healthcare institutions bore the responsibility for early detection, early treatment, and early intervention, serving as the linchpin in preventing sustained community transmission of the epidemic (4). Under government directives, primary healthcare institutions established a joint prevention and control system, horizontally coordinating with communities and street offices to provide basic medical and public health services to residents within their jurisdiction. Activities such as education and promotion, home isolation, and nucleic acid sampling constitute their pandemic prevention and control efforts. Vertically, they strengthened connections with higher-level healthcare institutions and disease control centers, strictly implementing the responsibility system for initial diagnosis. They ensured the smooth transfer of suspected fever patients and guide fever patients to seek medical treatment (5).

Primary healthcare workers played an essential role in public health crises, acting as the frontline workers of the public health system. Their responsibilities were not limited to routine diagnosis and treatment but also involved epidemic monitoring, case tracking, health education, and vaccination programs (6). In large cities like Guangzhou, they were the bridge connecting residents to higher-level medical services. Their work was related not only to individual health but also to the public health safety of the entire community and city.

Studies had shown that the rapid changes in the pandemic required primary healthcare workers to continuously update their knowledge and skills to adapt to new prevention and control guidelines and treatment methods (7). Furthermore, due to the uncertainty of the pandemic, they often had to make quick decisions under limited resources, which increased work pressure and occupational risks (8). Additionally, primary healthcare workers needed to address panic and misunderstandings from patients and the public, which demanded stronger communication skills and psychological support techniques (9). During the COVID-19 pandemic, anxiety and depression were widespread among primary healthcare workers (10). The prevalence of sleep disorders was higher among the frontline healthcare workers compared to the non-frontline and non-medical staff, while anxiety and depression were prevalent in the entire cohort (11).

This study utilized qualitative interview methods to explore the work experiences of primary healthcare workers in Guangzhou during the COVID-19 epidemic prevention and control period. The research aimed to identify the primary difficulties and challenges they faced in their professional duties, analyze the causes of these issues, and offer recommendations for improvement based on the findings of the investigation. Although the COVID-19 pandemic has ended, the weaknesses exposed in the epidemic prevention and control work during this period require our continuous reflection. The significance of this study lies not only in the difficulties encountered in grassroots epidemic prevention work during the COVID-19 period but also in gaining a better understanding of the complexity and challenges of epidemic prevention and control. It is essential to continuously summarize experiences and learn lessons to prepare for potential public health events that may arise in the future.

## 2 Materials and methods

### 2.1 Participants

The study participants were selected from primary healthcare institutions in Guangzhou City, based on specific inclusion criteria: (1) They have been involved in grassroots COVID-19 epidemic prevention and control work since June 2021 or even earlier, possessing extensive on-site experience and a profound understanding of grassroots epidemic prevention work; (2) Their involvement in grassroots epidemic prevention work includes activities such as nucleic acid sampling, household management, vaccine administration, transportation of high-risk individuals, operation of epidemic prevention systems, and three-person team investigations; (3) They provided informed consent and voluntarily participated in the study.

### 2.2 Sampling

Purposive sampling was used in this study to select eligible healthcare workers engaged in grassroots epidemic prevention from healthcare institutions. Across Tianhe, Baiyun, and Liwan districts. Tianhe, as the Central Business District (CBD) of Guangzhou City, was characterized by a high degree of urbanization and bustling commercial activities. With significant population mobility and frequent economic activities, this area faced unique challenges in epidemic prevention and control. Baiyun was one of the most populous areas in Guangzhou City. The dense population led to more complex demands for epidemic prevention and control. Liwan, an old town area of Guangzhou City, was the first area in Guangzhou to implement community-level epidemic lockdown and management measures, which provided it with unique experiences and lessons in epidemic prevention and control (12). The selected primary healthcare workers included a variety of professional types, such as general practitioners, public health physicians, nurses, and administrative personnel. The sample size was determined through information saturation (13, 14), where data collection proceeded until no new insights emerged from additional interviews.

### 2.3 Research design

This study utilized qualitative research methods, conducting semi-structured interviews with epidemic prevention personnel and special team members from primary-level medical and health institutions. The aim was to explore the status of grassroots epidemic prevention work, the existing problems and their impacts, the needs for protective measures for the epidemic prevention staff, and suggestions for the optimization of grassroots epidemic prevention efforts. Qualitative research data are collected through a variety of means, such as interviews, direct observations, personal narrative statements, documents, photographs, and audio or video recordings (15). Investigators have the flexibility to change the content and flow of questions when needed to explore deeper meanings (16). The use of qualitative research allowed for an understanding of new phenomena (17), which was crucial for mitigating the difficulties and challenges faced by primary healthcare workers in epidemic prevention work. The findings from the analysis called for action to be taken.

We applied the thematic analysis approach proposed by Braun and Clarke to identify themes from the collected data (18). Thematic Analysis is a qualitative research data analysis technique that involves searching within datasets to identify, analyze, and report recurring patterns known as themes. This method not only describes data but also performs interpretive work during the selection of codes and the construction of themes, demonstrating its flexibility and independence from specific epistemological frameworks. Braun and Clarke define a theme as a “patterned response or meaning,” distinguishing between latent and semantic themes. Semantic themes deal with explicit or surface meanings in the data, while latent themes reveal deeper implications, assumptions, or meaning formations. The six-step analysis method they propose is a nonlinear and recursive process that includes familiarizing oneself with the data, forming initial codes, searching for themes, reviewing themes, defining and naming themes, and writing the analytical report. Thematic Analysis is oriented towards addressing research questions. Although its process is similar to Grounded Theory, its core objective is not to find core concepts that reflect the essence of phenomena and construct related theories based on systematic data collection. Instead, it aims to identify and refine “themes” from raw data and use these themes to analyze research questions (19). Thematic Analysis differs from Content Analysis in methodology as well. Content Analysis systematically encodes, classifies, and semantically judges text content to analyze word frequency and relationships between terms, focusing on a more quantified approach to research questions (20). In contrast, Thematic Analysis does not solely rely on word frequency to identify “themes” but bases its analysis on the thematic relevance to the research issue, which may appear frequently or infrequently in the data.

To protect the rights and interests of interviewees, prior to conducting interviews, interviewers proactively inform participants that their contributions will remain anonymous and solely utilized for research purposes. It is ensured that the interviews do not violate any rights of the participants, who retain the right to voluntary participation, refusal, or withdrawal from the study. All interviews are conducted in familiar and quiet settings, with participants’ informed consent obtained before recording. Interviewers adhere to the original interview outline but also delve deeper into relevant topics based on participants’ responses. They promptly adjust questioning methods and sequences in response to interview data to ensure the collection of authentic and accurate information.

Prior to undertaking our research, our project team first engaged in direct participation, immersing ourselves in grassroots epidemic prevention work. This firsthand experience provided us with invaluable primary data and a profound understanding of the challenges faced at the grassroots level. Building upon this foundation, we conducted an extensive review of literature related to qualitative interviews and studied literature concerning the difficulties and challenges encountered in grassroots epidemic prevention work. Based on our practical experience and literature review, we formulated a detailed interview protocol. As detailed in Table 1.

**Table 1:** Semi-structured interview outline.

Questions
1. Could you please provide a brief introduction about yourself? For example, your age, educational background, work experience, etc.
2. What are the epidemic prevention and control tasks you have been involved in over the past year?
3. How do you allocate your time between work, particularly epidemic prevention, and your original duties? What aspect of epidemic prevention work consumes the most time? Is the workload evenly distributed among team members, and is there any quantitative representation of this distribution?
4. How has epidemic prevention work affected your daily tasks, such as clinic closures or suspension of departmental activities?
5. How do you balance epidemic prevention efforts with basic public health and medical services? What strategies can be implemented to optimize epidemic prevention policies and mechanisms and minimize their impact?
6. What challenges during epidemic prevention and control have affected your work and personal life? (For example, personnel, subsidies, resources, management, etc.)
7. What are your thoughts on the sustainability of epidemic prevention efforts?
8. What measures do you take to protect your physical and mental well-being as a healthcare worker?
9. Regarding improvements in epidemic prevention work at community health service centers, do you have any additional thoughts or suggestions?

### 2.4 Data collection

Interviews were conducted by two researchers (JYQ and FHY) in two-person teams, with one researcher taking the lead in guiding participants through the interviews, while the second researcher was responsible for taking field notes and prompting the participants when necessary. Before commencing the interview, the interviewer explained the interview’s purpose and content to the participant, confirmed the interview details including time and location, and proceeded with complete audio recording throughout the interview. Key points were also noted down in writing. Before the formal interview, 5–10 min were spent explaining in detail the purpose, content, and methods of the study, as well as confidentiality commitments, the voluntary nature of the research, and the required informed consent. The formal interview lasted approximately 45–60 min. Throughout the interview, appropriate communication techniques were employed, with the interviewer avoiding directive statements and actively encouraging the interviewee to freely articulate their perspectives. The interview questions and sequence were flexibly adjusted based on the interviewee’s responses and interests. Each participant was interviewed only once. To ensure the completeness and accuracy of the interview materials, the written record was returned to the participant for corrections at the end of the interview. On the same day the interview was conducted, the audio recording and notes were integrated to form textual content, which was then sent to the interviewee for review and final confirmation.

### 2.5 Data analysis

To ensure interviewees’ privacy, numerical codes are assigned sequentially based on interview time. Following interviews, recorded content is transcribed into text on the same day. We employed NVivo V.12 for the management, storage, and organization of all data. We scrutinized and analyzed the data using thematic analysis, identifying and extracting key themes through a rigorous structured analysis methodology that ensured a structured academic approach to thematic analysis. (1) Familiarization: The text is thoroughly reviewed multiple times to understand interviewee contributions. (2) Coding: Meaningful segments (e.g., phrases, sentences, paragraphs) relevant to research questions are identified and coded preliminarily. (3) Theme Development: Initial themes are derived from coded segments, and all relevant codes and excerpts are categorized under each theme. (4) Theme Review: Themes and corresponding excerpts are reviewed for consistency and completeness, ensuring no relevant codes are overlooked. (5) Refinement: Themes are further refined for clarity and coherence, organizing excerpts to construct cohesive narratives. (6) Reporting: Findings are summarized and documented in the final report. All interviews were transcribed verbatim by the first author (JYQ) and checked for accuracy and completeness by a second researcher (FHY). Triangulation was employed to ensure the credibility of the analysis. This involved randomly selecting 10% of the transcripts for independent analysis by researchers who were not involved in the data collection process. In cases of discrepancies, team meetings were frequently held to discuss and review until a consensus was reached. The sample became saturated after the 12th interview, and two additional interviews were conducted to ensure that no more data could be collected. No participants withdrew during the interview period.

### 2.6 Ethical considerations

The study was conducted according to the guidelines of the Declaration of Helsinki, and approved by the Institutional Review Board of Jinan University (NO.JNUKY-2023-0037). The study team obtained informed consent from all participants. Participants were made aware that they could withdraw at any time.

## 3 Results

### 3.1 Participant characteristics

In this study, a total of 14 healthcare workers from three districts of Guangzhou City, who had participated in grassroots epidemic prevention work, were interviewed. Among them, there were 7 males and 7 females, all with undergraduate degrees. Their positions included public health physicians, general practitioners, nursing management personnel, and hospital infection control department heads, among others. Please refer to Table 2.

**Table 2:** General information of interviewees.

Interview subjects	District	Gender	Age	Position or job title	Participated in epidemic prevention work
P1	Tianhe	Male	27	Head of infectious disease control and prevention	Interpretation of new policies, epidemic prevention Q&A, information officer
P2	Tianhe	Female	27	Public health physician	Door-to-door sampling, large-scale sampling, transport of close contacts to quarantine hotels
P3	Tianhe	Male	35	Preventive health leader	Three-person team work and transportation of close contacts with high transmission
P4	Tianhe	Male	41	General physician	Sampling, vaccination, hotel accommodation and transportation, fever management
P5	Tianhe	Female	35	Nursing and infection control manager	Infection control work in epidemic prevention and control, logistics support work
P6	Tianhe	Female	34	Rehabilitation and epidemic manager	Coordinating and arranging sampling work, handling sampling information
P7	Tianhe	Female	38	HR manager	Assisting units with nucleic acid sampling order
P8	Tianhe	Male	28	Full-time epidemic manager	Coordination and scheduling of three-person team work, information coordination and submission
P9	Baiyun	Male	36	Preventive health director	Coordination and personnel scheduling for three-person team community prevention and control work
P10	Baiyun	Male	33	Public health physician	Hotel investigation, outbound investigation by borrowed staff
P11	Baiyun	Female	37	Infection control director	Hotel investigation, outbound investigation by borrowed staff
P12	Liwan	Female	26	Epidemic management leader	Epidemic prevention Q&A, community information coordination and integration submission
P13	Liwan	Male	35	Public health information officer	Community information coordination and integration submission
P14	Liwan	Female	32	Public health physician	Nucleic acid sampling, transport of close contacts to quarantine hotels

### 3.2 Dimensions of challenges in grassroots epidemic prevention work

By organizing and summarizing the interview results, the difficulties in grassroots epidemic prevention work were primarily categorized into seven dimensions: human resource allocation, material and vehicle support, epidemic prevention subsidies and compensation, humanistic care, upper-level management, horizontal collaboration, and resident cooperation. Please refer to Table 3.

**Table 3:** Initial coding, key themes, and overall themes of theme analysis.

Overall themes	Subthemes	Initial coding
Human resource allocation	1. Insufficient personnel numbers	Loss of non-staff personnel; Insufficient manpower lead to unreasonable work and rest schedules
	2. Imbalanced personnel structure	Public health personnel are more prone to attrition than clinical staff; Imbalance in the proportion of internal and external staff; Establishment numbers not meeting the corresponding ratio to service personnel
	3. Unreasonable work allocation	Mismatch between the number of workers and workload; Individuals capabilities do not match job requirements; Heavy burden of epidemic prevention tasks for certain positions
	4. Need for improvement in competency	Inconsistent work abilities; Inadequate training methods; Training course content is overly intricate
Material and vehicle support	1. Inadequate integration and management of supplies	Unclear storage locations for supplies; Shortages discovered only when needed; Inadequate integration and management
	2. Insufficient Vehicles for Dispatch	Insufficient unit vehicles; Relying on taxis for external work
Epidemic prevention subsidies and compensation	1. Insufficient subsidies	Subsidies not distributed according to work performed
	2. Imperfect subsidy mechanism	Skewed subsidy allocation; Untimely disbursement; Inability to compensate for missed breaks with other forms of subsidy
Humanistic care	1. Lack of institutional support	Lack of humanitarian care; Neglect of individual circumstances; Importance of fostering a conducive work atmosphere and culture
	2. Lack of family support	Discontent from family members due to late-night work; Inability to fulfill family responsibilities
	3. Lack of social support	Epidemic prevention requires support from all sectors of society; Society lacks a respectful and supportive atmosphere for epidemic prevention workers
Upper-level management	1. Issues with policy measures	Unclear division of responsibilities; Failure to implement epidemic prevention plans in actual practice
	2. Problems with inspection and supervision	During severe epidemics, there is a need to welcome more inspections; frequent inspections; Redundant data submissions; Excessive documentation
Horizontal collaboration	1. Police	Not every police officer is willing to assist in door-to-door visits or transportation tasks
	2. Resident committee	Resident committees need to lead the organization of large-scale nucleic acid sampling
Resident cooperation	1. Low level of resident cooperation	Residents' lack of cooperation results in spending double the time; Dissatisfaction with pre-screening and triage in outpatient epidemic prevention work; Rejection of some mandatory quarantine measures

#### 3.2.1 Theme 1: human resource allocation

##### 3.2.1.1 Sub-theme 1: insufficient personnel numbers

The grassroots epidemic prevention work, due to its heavy and complex nature, demands a significant amount of human resources. However, the reality faced by the majority of grassroots epidemic prevention personnel was a shortage of workers. This shortage forced the remaining workers to take on a workload beyond the ordinary, leading to a sharp increase in their work intensity and prolonged exposure to a high-pressure work environment. This state of overload not only posed a threat to the health and well-being of individuals but also severely affected work efficiency and service quality. What was even more serious was that the heavy burden of epidemic prevention caused by the personnel gap made the remaining workers more likely to choose to leave when facing sustained work pressure, thereby exacerbating the problem of personnel turnover. The departure of personnel not only weakened the strength of grassroots epidemic prevention but also further expanded the personnel gap, creating a vicious cycle.

*“Staff attrition, especially non-official personnel leaving, many have departed, and even if new ones arrive, they soon leave as well”* (P1).

*“The most prominent issue is the shortage of manpower, which leads to unreasonable scheduling of work and rest time”* (P3).

*“Staff attrition can be severe, especially in public health”* (P4).

*“The shortage of manpower is not only a problem in our unit, but in fact, every grassroots unit lacks sufficient personnel”* (P9).

##### 3.2.1.2 Sub-theme 2: imbalanced personnel structure

Primary healthcare institutions serve as the cornerstone of the public health system, and the services they provide are directly linked to the health and well-being of community residents. If these institutions faced a shortage of healthcare personnel in specific professional fields, it would severely impact the quality and efficiency of grassroots health services, thereby affecting the overall epidemic prevention status of the community. Furthermore, when there was an insufficiency of workers in certain professional fields, healthcare workers from other fields might have had to bear additional workloads to compensate for the service gaps.

*“The lack of specialized public health physicians and insufficient manpower result in a significant proportion of frontline clinical medical staff participating in epidemic control, thereby impeding the effective delivery of medical services”* (P4).

*“An uneven personnel structure affects the entire department, including scheduling at the center and other work arrangements”* (P5).

##### 3.2.1.3 Sub-theme 3: unreasonable work allocation

There was a mismatch between the number of personnel and the workload, which led to a decline in work efficiency and service quality. Specifically, there was an insufficient number of workers for heavy-duty tasks, while there was an excess for simpler tasks. This imbalanced allocation severely affected the progress and effectiveness of the overall work. Additionally, the issue of personnel capabilities not aligning with the nature of the work could not be overlooked. If the professional skills and experience of the workers did not match the tasks they undertook, it would be difficult for them to perform at their best, thereby affecting the progress and outcomes of the work. The distribution of epidemic prevention tasks was overly skewed towards some primary healthcare workers, which not only subjected them to unreasonable work burdens but also hindered the effective conduct of their primary duties.

*“Almost all epidemic prevention tasks require public health personnel to be dispatched because we do not need to run outpatient clinics, so there will definitely be more epidemic prevention shifts”* (P2).

*“The epidemic control consumes most of our time and manpower, resulting in a lag in public health work, yet the targets from higher-ups have not decreased but increased”* (P3).

*“Some positions require young computer-savvy individuals to work fast, but sometimes older individuals are assigned computer tasks”* (P12).

*“Many non-medical technical issues are handled by medical personnel, with excessive paperwork consuming manpower for management and organization”* (P13).

##### 3.2.1.4 Sub-theme 4: need for improvement in competency

As the frontline warriors in the fight against the pandemic, the learning ability and professional literacy of primary healthcare workers were key to ensuring the smooth progress of epidemic control work. In the ever-changing environment of the epidemic, these capabilities were particularly crucial. Only with the ability to quickly adapt to and respond to new situations, coupled with solid professional literacy, could primary healthcare workers properly handle the heavy and complex tasks of grassroots epidemic prevention. However, in the face of intense and heavy work tasks, the issues of insufficient capability and professional training among some workers gradually emerged. These issues not only affected the efficiency and quality of epidemic prevention work but also had adverse effects on the career development and mental health of the workers themselves.

*“There is disparity in work capabilities. Some individuals simply do not grasp it, no matter how much you teach them, while others understand it instantly”* (P1).

*“These matters are specialized. They cannot be understood with just a few words”* (P3).

*“There are some online training sessions, but in reality, few people attend them because everyone is very busy. Many individuals initially performing nucleic acid sampling at the grassroots level do not follow standardized procedures”* (P7).

#### 3.2.2 Theme 2: material and vehicle support

##### 3.2.2.1 Sub-theme 1: inadequate integration and management of supplies

To ensure the smooth progress of grassroots epidemic prevention work, it was imperative for primary-level medical and health institutions to stock up on ample epidemic prevention supplies. However, merely stockpiling supplies was not sufficient; effective integration and management of these supplies were crucial for enhancing the efficiency of usage and ensuring the efficient conduct of epidemic prevention work. At present, some units had issues with supply management that urgently needed to be addressed. During an outbreak, it was vital to quickly and accurately allocate supplies to where they were needed. If the information on supplies was unclear, the inventory data was inaccurate, or the allocation process was complex, it could delay the distribution of epidemic prevention supplies, affecting the timeliness and effectiveness of epidemic prevention work.

*“Supplies are not effectively integrated, and sometimes it’s only when needed that we realize they are not available, as there is no designated personnel to organize them”* (P2).

*“Sometimes colleagues return from work and just randomly place supplies in the room. Despite having personnel assigned to regular cleanup, it still remains disorderly”* (P10).

*“Supplies are not effectively arranged. They are just categorized and placed aside. There is no dedicated public health emergency warehouse”* (P11).

##### 3.2.2.2 Sub-theme 2: insufficient vehicles for dispatch

The epidemic prevention work of primary healthcare workers not only included door-to-door investigation and sampling for the homebound population but also involved real-time monitoring of the epidemic and rapid response. In this process, the guarantee of transportation was one of the key factors to ensure the smooth progress of epidemic prevention work. Insufficient transportation support directly affected the efficiency of epidemic prevention work. If transportation facilities were inconvenient, it would greatly increase their travel time, reduce work efficiency, and even possibly miss the best opportunity for epidemic prevention. However, this need was often overlooked in actual operations.

*“We have insufficient vehicles. Often, when going out for work, we need to take taxis ourselves, which is very inconvenient”* (P1).

*“The main issue is the shortage of vehicles. Routine tasks and emergency support cannot be balanced”* (P4).

*“Vehicle use is indeed a problem. Sometimes, our unit’s vehicles cannot even be coordinated. Just because the unit has a car does not mean it can be used directly for you.”* (P14).

#### 3.2.3 Theme 3: epidemic prevention subsidies and compensation

##### 3.2.3.1 Sub-theme 1: insufficient subsidies

Primary healthcare workers played an irreplaceable role on the frontlines of combating the epidemic, bearing immense work pressure and occupational risks. After completing a large amount of epidemic prevention work, reasonable subsidies and compensation were crucial for enhancing their work enthusiasm, safeguarding their rights and interests, and motivating their continued commitment to epidemic prevention. However, there were still issues where some workers did not receive the due subsidies or did not receive compensation according to their labor, and economic uncertainty might have made it difficult for them to fully commit to their work, affecting the quality and efficiency of their work. This not only affected their motivation but also posed a challenge to the long-term effectiveness of grassroots epidemic prevention work.

*“There are no epidemic prevention allowances for those of us who are in official positions. There might be some for non-official positions, but we still cannot retain personnel, indicating that the epidemic prevention subsidy is indeed very minimal”* (P1).

*“We have subsidies, but I think they are not substantial”* (P2).

*“We are in official positions, so it’s okay, but for those who are not, the pay is not much. Indeed, because of this issue, we have had at least five or six people resign”* (P11).

*“The workload is high, but the subsidies are minimal”* (P14).

##### 3.2.3.2 Sub-theme 2: imperfect subsidy mechanism

The epidemic prevention subsidy mechanism was an essential safeguard to ensure that primary healthcare workers received necessary support and incentives when facing the challenges of the epidemic. A robust subsidy system could not only provide them with necessary financial support but also reflect recognition and respect for their hard work. However, due to the imperfections in the subsidy mechanism at the time, there were some urgent issues in the distribution and implementation of epidemic prevention subsidies, such as biased distribution of subsidies, delays in disbursement, and the inability to convert untaken leave into other forms of compensation.

*“The subsidies are not distributed promptly. It’s usually delayed by half a year or even a year”* (P2).

*“Official personnel cannot take compensatory leave, and it cannot be exchanged for other allowances”* (P4).

*“For official personnel, there is basically no epidemic prevention subsidy for outbound sampling or vaccine administration, but temporary hires may receive it. The subsidy policy is also not very comprehensive, and the disbursement is not timely enough”* (P10).

#### 3.2.4 Theme 4: humanistic care

##### 3.2.4.1 Sub-theme 1: lack of institutional support

After engaging in long-term epidemic prevention and control work, primary healthcare workers not only faced physical fatigue but also had to cope with psychological stress. Under such circumstances, the support from their organization became a crucial factor in enabling primary healthcare workers to continue their efforts. However, numerous issues had persisted in various aspects such as work management systems, personal development opportunities, welfare benefits, and physical and mental health care.

*“The arrangement of work and rest time is unreasonable because time is spent on what I personally consider meaningless tasks”* (P1).

*“The compensatory leave system urgently needs improvement, as it is vital for safeguarding the lives of grassroots epidemic prevention workers”* (P4).

*“There should be an increase in welfare subsidies and opportunities for civil service examination, especially for contractual employees”* (P6).

*“During maternity leave, there is no compensation, indicating inadequate humanistic care”* (P14).

##### 3.2.4.2 Sub-theme 2: lack of family support

Primary healthcare workers not only bore the heavy burden of grassroots epidemic prevention work but also almost every individual shoulders their family responsibilities. When work responsibilities consumed most of one’s time and energy, solving family concerns became a challenge. The prolonged work and constant state of readiness made it difficult for grassroots epidemic prevention personnel to devote sufficient time and energy to their family life. This not only affected the time they spent with their family but could also lead to emotional estrangement among family members, increasing the stress on family relationships.

*“Physical fitness declines, leading to anxiety, and nighttime work outings cause dissatisfaction among family members”* (P1).

*“There’s conflict between work and family. Even when working from home, there’s no time to take care of the children”* (P3).

*“There’s conflict between work and family. Since the outbreak of the epidemic, I have not been home for major holidays”* (P10).

*“In dual-income families, both parents are on assignment, leaving no one to care for the children”* (P11).

##### 3.2.4.3 Sub-theme 3: lack of social support

The importance of grassroots epidemic prevention work often exceeded the general public’s understanding. This job not only required primary healthcare workers to have professional knowledge and skills, but also demanded a significant investment of energy and emotion, facing high-intensity work pressure and complex work situations. However, when this heavy responsibility and hard work did not receive widespread understanding and support from society, some primary healthcare workers might have felt even more physically and mentally exhausted. This situation reflected a deficiency in the support and encouragement of grassroots epidemic prevention personnel by current social opinion and relevant social departments.

*“Epidemic prevention work is overwhelming, leading to significant psychological stress and a lack of understanding from others”* (P2).

*“I hope that grassroots unions can offer diverse forms of support and care”* (P5).

*“I hope that teachers in schools can pay attention to students’ studies. Because during the epidemic, there has not been much time to focus on children’s learning”* (P7).

#### 3.2.5 Theme 5: upper-level management

##### 3.2.5.1 Sub-theme 1: issues with policy measures

The continuous evolution of the epidemic posed new challenges to epidemic prevention work, requiring policy measures to be updated in a timely manner to adapt to new situations. However, primary healthcare workers sometimes encountered problems with policy measures when executing their tasks, which affected the efficiency and effectiveness of the epidemic prevention work. The ambiguity of work guidelines brought operational confusion to primary healthcare workers. Epidemic prevention measures required specific and clear guidance so that workers could execute them swiftly and accurately. The vagueness of the guidelines not only increased the difficulty of execution but could also lead to the inadequate implementation of epidemic prevention measures, affecting the effectiveness of control. Moreover, in epidemic prevention work, every link was crucial, requiring clear responsibility allocation to ensure that every worker was aware of their duties and tasks. Unclear division of responsibilities could lead to overlapping or omission of work, affecting the coordination and coherence of the overall epidemic prevention work.

*“Superiors often distribute documents without thoroughly reading and understanding them”* (P1).

*“When problems arise in epidemic prevention work, it’s difficult to identify the responsible unit or individual because the documents lack specificity and the division of responsibilities is unclear”* (P4).

*“Epidemic prevention measures are continually intensified without clear benchmarks. Without clear guidance at the grassroots level, it’s challenging to implement control measures”* (P8).

*“While there are overarching frameworks and documents, each individual’s interpretation of the documents may* var*y”* (P13).

##### 3.2.5.2 Sub-theme 3: problems with inspection and supervision

Inspection and supervision were key to ensuring the efficiency and orderliness of grassroots epidemic prevention work, playing an irreplaceable role in identifying problems promptly, guiding improvement measures, and enhancing work quality. However, when inspection and supervision became too frequent or overly formal, it required primary healthcare workers to spend a significant amount of time preparing for and participating in inspections, potentially becoming a source of work burden and stress for them, thereby diverting their attention and energy from the core epidemic prevention tasks.

*“Sometimes, there are overlapping inspections, with vertical supervision from superiors, as well as inspections between districts, but each expert who comes gives different opinions”* (P5).

*“There are many inspections. They occur frequently, every few days”* (P7).

*“During periods of stable epidemic conditions, inspections and supervision might decrease, but when there is a local outbreak, there are inspections every day”* (P11).

*“Different higher-level units require the completion of reports, but some of the content is repetitive, resulting in redundant efforts by personnel to fill out forms”* (P13).

#### 3.2.6 Theme 6: horizontal collaboration

Grassroots epidemic prevention work was a systematic project, relying on the close collaboration and joint efforts of various parties such as primary-level medical institutions, community neighborhood committees, and public security departments. However, in the actual operation process, issues in horizontal collaboration, such as low levels of coordination among members, could become factors hindering the smooth progress of epidemic prevention work. In epidemic prevention, a swift and unified response was key to controlling the spread of the epidemic. If there was a lack of efficient coordination and communication among the three-person team members, it would be difficult to achieve rapid response and unified action.

*“Some police officers are unwilling to cooperate in conducting door-to-door tasks. When encountering residents unwilling to cooperate with transportation, some police officers also refuse to assist”* (P2).

*“The responsibilities of the three-person teams are not effectively implemented. Many tasks that could be completed by community committees and police officers are instead assigned to community health workers”* (P3).

*“Without support from police officers, the three-person teams often face resistance and lack of cooperation from residents during door-to-door checks and sampling”* (P10).

*“Community health workers are burdened with too many responsibilities within the three-person teams”* (P14).

#### 3.2.7 Theme 7: resident cooperation

The core of grassroots epidemic prevention work lay in the extensive participation and active cooperation of residents within the jurisdiction, as their understanding and cooperation were key to implementing effective epidemic prevention measures. However, in actual work, primary healthcare workers sometimes encountered residents who did not understand or cooperate, which not only increased the difficulty of epidemic prevention work but could also have a negative impact on the effectiveness of prevention. A lack of understanding from residents could lead to the obstruction of the dissemination of epidemic prevention information. If residents lacked awareness of the necessity and urgency of epidemic prevention measures, they might not take epidemic prevention guidance and advice seriously, thereby affecting the implementation of these measures. Faced with uncooperative residents, primary healthcare workers needed to spend more time and energy explaining, persuading, and educating these residents.

*“The majority of residents are cooperative, but there are still a few who are not cooperative”* (P1).

*“Most residents are not rejecting, but there are indeed some cases where they refuse to cooperate. Sometimes, even when we are prepared, residents refuse to come out of their homes to cooperate”* (P2).

*“Conducting screenings is extremely difficult. Some residents may even lodge complaints”* (P10).

*“There are some who are unwilling to receive vaccinations”* (P12).

## 4 Discussion and recommendations

### 4.1 Discussion

In this study, interviewees indicated that insufficient personnel is a critical factor affecting the implementation of epidemic prevention work and is the fundamental reason for the unreasonable scheduling of work and rest time. Furthermore, an imbalance in personnel structure, with shortages of certain specialized personnel, necessitates the substitution of other professionals, making it difficult to carry out their primary duties. Previous studies have found that some regions still face issues of imbalanced development, unreasonable allocation, and low quality of primary healthcare human resources (21, 22). Following the outbreak of the epidemic, the proportion of grassroots public health professionals in Tongzhou District, Beijing, decreased, reflecting the loss and insufficient introduction of grassroots public health talents in the region, which has been the trend in healthcare talent development in the area over the past decade (22). Whether before or after the epidemic, in urban or rural areas, the shortage of grassroots public health talent remains a prominent issue contributing to the imbalance in the structure of healthcare personnel (23–25). Additionally, epidemic prevention work required collaboration among multiple individuals, and when personnel capabilities did not align with the nature of the work, the efficiency of epidemic prevention work was significantly affected. Inadequate infectious disease prevention and control capabilities had already been present in some primary healthcare institutions, and with the increase in COVID-19 prevention and control efforts and the development and utilization of new healthcare systems, further inadequacies in the professional competence and training of primary healthcare personnel were exposed.

At the beginning of the COVID-19 pandemic, the shortage of primary healthcare supplies was the primary problem. As the problem of insufficient material allocation was gradually alleviated, the importance of material management issues gradually came to the fore. Ensuring that grassroots emergency supplies can be rapidly deployed and utilized is the key to improving the management level of public health emergency supplies stockpile, and is also an important guarantee to enhance the ability of grassroots to respond to public health emergencies (26). In the process of fighting the COVID-19 epidemic, the imperfections of the grassroots emergency material reserve system and the low level of material management informatization were exposed due to the suddenness and urgency of the task. Many primary healthcare institutions still used the traditional warehouse management method, and the inventory and registration of materials rely on manual operation, which not only consumed manpower, but also affected the accuracy and efficiency of management (27). Due to the lack of an effective monitoring mechanism, materials might not have been replenished in a timely manner when they were out of stock, or might have been requisitioned without being put into storage in a timely manner, which affected the operational efficiency of the organization and the efficiency of access to materials (28).

In the early stages of the COVID-19 outbreak, the Central Leading Group for COVID-19 Response issued the “Notice on Fully Implementing Measures to Further Protect, Care for, and Support Medical Personnel,” which explicitly outlined specific requirements such as “localities must issue temporary work subsidies to medical personnel involved in epidemic prevention and control, increase the total amount of one-time performance pay, and implement healthcare and epidemic prevention allowances for healthcare personnel” (29). Due to the delayed nature of subsidies, it was possible that certain regions faced constraints due to economic development or institutional financial conditions, resulting in the delayed distribution of subsidies. The epidemic prevention subsidy policy directly impacts the interests of each prevention and control worker and plays a significant motivating role. If compensation subsidies do not accurately reflect the labor value of individuals, it can significantly impact the job satisfaction and motivation of healthcare personnel (30).

During the COVID-19 pandemic, government departments at all levels responded swiftly, adopting a top-down task assignment model for epidemic prevention and control to ensure the mandatory distribution and implementation of epidemic prevention tasks. This model played a crucial role in rapidly mobilizing resources and manpower. However, due to the suddenness and novelty of the outbreak, even senior leaders may have faced challenges due to lack of experience, which to some extent led to deficiencies and vulnerabilities in management. Amidst the rapid development of the epidemic and the continuous changes in policies, guidance for grassroots epidemic prevention work might become unclear, and the division of responsibilities might become ambiguous, leading to inconsistencies between actual execution and higher-level policies. Primary healthcare workers sometimes did not clearly understand their specific tasks, and community residents were uncertain whom to turn to for questions about epidemic prevention measures. Moreover, due to the lack of targeted measures, the top-down task assignment sometimes tended to adopt a “one-size-fits-all” or “increasingly stringent” approach, which not only restricted the effectiveness of grassroots prevention work but also increased the burden on Primary healthcare workers and the inconvenience to the public (31). The frequency of higher-level inspections was partly due to a close watch on the implementation of prevention and control measures, as well as a high level of concern for the development of the epidemic. However, if there was a lack of effective communication between higher authorities, it might have led to repetitive or excessive inspections, which not only consumed grassroots resources and energy but might also interfere with normal epidemic prevention work. Phenomena of bureaucracy and formalism, such as turning “information reporting” into a formalized process, failed to truly serve the purpose of prevention and control, instead adding to the burden on the grassroots level (32). Under the influence of the trace management philosophy, there might have been a tendency in the epidemic prevention process to focus more on evidence than actual effectiveness. This led primary healthcare workers to spend a lot of time preparing for inspections and leaving records, which in turn affected their focus on the prevention and control work itself. Such a tendency might have caused grassroots epidemic prevention personnel to work for the sake of coping with inspections, rather than for the actual effect of prevention and control, which contradicted the original intention of the inspections and supervision (33).

From the interviews, it can be observed that there were other issues affecting the implementation of grassroots epidemic prevention work, such as inadequate coordination among the three-person teams and insufficient cooperation from residents. However, it was noted in the interviews that only a small number of police officers or community workers failed to fulfill their responsibilities, thereby reducing the effectiveness of grassroots epidemic prevention work. Additionally, the majority of residents understood and cooperated with the epidemic prevention efforts, with only a few residents showing resistance. While these situations were difficult to completely avoid, efforts can be made to enhance publicity, change perceptions, and increase cooperation.

### 4.2 Recommendations

#### 4.2.1 Strengthening the allocation of primary healthcare workers and optimizing personnel management

While the occurrence of epidemics remains unpredictable, it is crucial to promptly assess current issues in epidemic prevention efforts and address them proactively to glean valuable insights for effectively managing potential future epidemics. The emergence of the COVID-19 pandemic has underscored the pivotal role of grassroots efforts in epidemic prevention and control, particularly within a well-functioning grassroots public health system. Therefore, enhancing the workforce at the grassroots level, particularly through robust training and recruitment of public health professionals, is essential. Establishing corresponding human resource allocation plans and coordinating work reasonably will ensure a more balanced distribution of talent. It is crucial to implement proper rotation management for staff, ensuring an adequate pool of backup personnel for different positions to prevent prolonged work periods for primary healthcare workers during times of heightened epidemic activity. Timely training for new recruits should focus on enhancing medical skills and standardizing learning processes, with an emphasis on emergency response and epidemic data management training for grassroots personnel, while reducing non-medical skills training that may be less relevant (34).

#### 4.2.2 Strengthening the emergency supplies reserve management mechanism and optimize logistical resource allocation

Strengthen the establishment of emergency material management systems, assign dedicated personnel to manage materials, and ensure that materials are properly categorized, accessed according to regulations, and managed effectively. Provide regular training for material management staff to enhance their operational capabilities and professional qualities, ensuring proficiency in material management knowledge and skills. Define the responsibilities of material management personnel, and intensify supervision and assessment to ensure the regulation and effectiveness of material management. Improve the informatization management of emergency prevention and control materials by establishing an inventory information system for specialized management of material reception, distribution, and storage. Establish standardized protocols for emergency vehicle support, with institutions potentially dedicating specific emergency vehicles or collaborating with local communities to provide such resources. Alternatively, explore partnerships with third-party companies capable of supplying vehicles and drivers as needed.

#### 4.2.3 Improving incentive and welfare mechanisms to boost the motivation of primary healthcare workers

Establishing a dedicated fund for major epidemic prevention and control efforts can bolster emergency support capabilities. Governments at all levels may allocate a specific portion of their budget expenditures to establish an epidemic prevention special fund, managed in a fund-style manner with surplus funds reserved for future needs. This ensures a stable financial resource for responding to sudden public health emergencies and safeguarding epidemic prevention subsidies for frontline workers (35). Enhancing incentive and support policies is crucial to ensure that all personnel engaged in epidemic prevention and control receive appropriate subsidies. Comprehensive subsidy standards should be developed based on factors such as workload, working hours, job complexity, and risk level during epidemic prevention and control activities. This approach ensures that subsidies are distributed in a reasonable, equitable, and transparent manner based on justified criteria.

#### 4.2.4 Enhancing humanistic care support to improve the job satisfaction of primary healthcare workers

Facing the heavy tasks of grassroots epidemic prevention, it is particularly important to ensure that primary healthcare workers receive proper rest and support in a high-pressure working environment. Although they cannot leave their posts at will, institutions should take positive measures to reasonably arrange rest time and, where possible, establish dedicated rest areas to ensure that primary healthcare workers can get enough rest and a comfortable resting environment, thus avoiding over-fatigue caused by long working hours (36). To alleviate the pressure of primary healthcare workers’ family life, institutions can optimize and adjust the scheduling system in a timely manner according to the actual situation of the department and the age and family status of the primary healthcare workers. Such flexible arrangements can allow them to reduce worries about family while working and focus more on their work. In addition, career development is a matter of great concern to primary healthcare workers. After the epidemic, relevant departments and units should consider optimizing the promotion channels for primary healthcare workers. Targeted promotion plans can be formulated according to different professions and professional titles, and professional work knowledge and practical experience can be taken as the main content of the review. This will help to reduce the pressure of scientific research topics and papers that primary healthcare workers face in the promotion process (37). With the continuous deepening of medical system reform, the pressure faced by primary healthcare workers is also increasing. Therefore, managers should pay more attention to the humanistic care of primary healthcare workers, provide necessary support and help by listening to their needs and expectations. This kind of care can not only improve the satisfaction of primary healthcare workers with managers, but also effectively improve the overall efficiency and quality of primary healthcare work (33).

#### 4.2.5 Optimizing the upper-level management system to alleviate the workload of primary healthcare workers

To improve this situation, higher-level authorities need to enhance internal communication to avoid unnecessary repetitive inspections while ensuring that inspections are targeted and effective. In addition, processes should be streamlined to reduce the burden of formalism, allowing primary healthcare workers to focus their energy on the implementation and effectiveness of prevention measures. Through these measures, the efficiency and effectiveness of prevention work can be improved, while also reducing the pressure on primary healthcare workers and enhancing the satisfaction and trust of the public.

### 4.3 Limitations

Our study had several limitations. As with other qualitative studies, the perspectives and beliefs of the research subjects influenced the research process (38). However, the differing viewpoints of the researchers facilitated rigorous debate and data analysis to ensure appropriate generalization and thematic emergence. The sample size was relatively small, but the interview content reached saturation from the responses of the interviewees. This met the qualitative analysis needs of our study. The study was conducted in Guangzhou, and the difficulties in epidemic prevention work in different regions and cities may differ from those reported in Guangzhou. Therefore, caution should be exercised when applying the results of this study to other regions or countries.

## 5 Conclusion

Overall, this study found that during the COVID-19 pandemic prevention and control period, primary healthcare workers faced seven main challenges in carrying out epidemic prevention work, namely: manpower allocation, material and vehicle support, epidemic prevention subsidies and compensation, humanitarian care, superior management, horizontal cooperation, and resident cooperation. These issues not only affected the efficiency and quality of epidemic prevention work but also reflected the vulnerability of grassroots medical and health systems in responding to public health emergencies. The study suggests that efforts should focus on enhancing the professional competence of grassroots medical and health personnel, improving material management practices, establishing effective incentive and support mechanisms, enhancing humanitarian care, and optimizing superior management systems. These measures aim to comprehensively enhance the epidemic prevention capabilities and efficiency of grassroots medical and health institutions. They also seek to minimize obstacles and challenges faced by primary healthcare workers in conducting epidemic prevention work, alleviate their workload, and bolster their work motivation.

## Data Availability

The raw data supporting the conclusions of this article will be made available by the authors, without undue reservation.
